# Evaluation of a Change Detection Methodology by Means of Binary Thresholding Algorithms and Informational Fusion Processes

**DOI:** 10.3390/s120303528

**Published:** 2012-03-13

**Authors:** Iñigo Molina, Estibaliz Martinez, Agueda Arquero, Gonzalo Pajares, Javier Sanchez

**Affiliations:** 1 ETSITGC, Universidad Politécnica de Madrid, Campus SUR, Ctra. de Valencia, km.7, 28031 Madrid, Spain; 2 Faculty of Computer Sciences, Universidad Politécnica de Madrid, Campus de Montegancedo, 28660 Boadilla del Monte Madrid, Spain; E-Mails: emartinez@fi.upm.es (E.M.); aarquero@fi.upm.es (A.A.); jarsahe@hotmail.com (J.S.); 3 Faculty of Computer Sciences, Universidad Complutense Madrid, 28040 Madrid, Spain E-Mail: pajares@fdi.ucm.es

**Keywords:** optical sensors, change detection, thresholding, information fusion, ROC space

## Abstract

Landcover is subject to continuous changes on a wide variety of temporal and spatial scales. Those changes produce significant effects in human and natural activities. Maintaining an updated spatial database with the occurred changes allows a better monitoring of the Earth’s resources and management of the environment. Change detection (CD) techniques using images from different sensors, such as satellite imagery, aerial photographs, *etc.*, have proven to be suitable and secure data sources from which updated information can be extracted efficiently, so that changes can also be inventoried and monitored. In this paper, a multisource CD methodology for multiresolution datasets is applied. First, different change indices are processed, then different thresholding algorithms for change/no_change are applied to these indices in order to better estimate the statistical parameters of these categories, finally the indices are integrated into a change detection multisource fusion process, which allows generating a single CD result from several combination of indices. This methodology has been applied to datasets with different spectral and spatial resolution properties. Then, the obtained results are evaluated by means of a quality control analysis, as well as with complementary graphical representations. The suggested methodology has also been proved efficiently for identifying the change detection index with the higher contribution.

## 1. Introduction

For several decades, change detection (CD) has been usually applied in different scientific or engineering topics. For instance, in cartography, it arises from the need for updating spatial databases. Likewise, for environmental analysis and assessment, the detection of the different processes occurred on a territory are frequently required. Due to human activity and natural disasters, different regions around the world have experienced rapid changes affecting wide areas on the Earth surface, resulting in phenomena such as erosion, runoff and flooding, increases in CO_2_ concentration, climate change and biodiversity decline [1,2]. Given the wide and complex implications produced by these changes, academic institutions, government agencies and environmental organizations concerned with the management of natural resources around the world, consider of crucial importance monitoring and detecting these changes. Hence, ensuring the correct update of land cover maps is a necessary tool for an effective management of the natural resources, and an efficient urban and regional planning.

In Earth observation applications, CD has been traditionally performed using more or less sophisticated procedures and methods, based on panchromatic or multispectral optical images which have been acquired on different dates, and characterized by their spatial and spectral properties. In a CD process, the basic aim consists in detecting groups of “significantly different” numerical values among a set of registered images of the same geographical area. As a result of the increasing growth of research work on this particular subject, different CD methodologies have been established, which use multitemporal images supplied by sensors generally located on artificial satellites, as a primary source of information [3–6]. In these studies, CD analysis has been carried out exclusively on the basis of a single change indicator, either the difference index in the case of one single band images or the Change Vector Analysis (CVA) procedure for multispectral type images. For the latter, it is also possible to derive indicators based on the calculation of spectral indices, such as the Normalized Difference Vegetation Index (NDVI) [7]. On the other hand, Lu *et al.* [8] also introduced another set of change indices, which are also applicable to panchromatic or multispectral remote sensing images. Recently, CD indices as the “log-cummulants” [9] or the Mean Ratio Detector (MRD) [10] have been applied to Synthetic Aperture Radar (SAR) images in change detection processes. Moreover, in the case of very high resolution images, Sjahputera *et al.* [11] also suggest to compute textural features before deriving the corresponding change detection index. Le Hégarat-Mascle *et al.* [12] present a different set of change detection indices and suggest their simultaneous use in order to take profit of the exhibited individual qualities. Then, a final change document is derived through a multisource fusion process based on an evidential reasoning theory. However, the contribution of the different CD indices to the end result is not specified in this work.

Once these indices have been processed, the next step is focused on the estimation of the parameters that characterize the change/no-change categories contained in this set of images, so that the detection process might be optimized by means of specific decision rules, where the quality of the derived change detection map will depend directly on the established threshold values for these classes. This thresholding process may be controlled by an analyst, who selects the threshold interactively. While the outcome of this operation might provide acceptable results, this process remains supervised. However, for certain purposes such as the large amount of available data, every day more abundant due to the increasing number of remote sensing devices, it is recommended to apply a fully automatic or non-supervised procedure. It prevents fatigue in humans and minimizes possible errors derived from it. This problem can be solved using automatic binary thresholding procedures, which have proved to be particularly useful and accurate in presence of unimodal distributions [13], which is also the case in this study for the derived change detection indices. Some of these methods include iterative, clustering and entropy based algorithms, which have demonstrated their effectiveness and applicability in the field of image thresholding [14]. In the case of CD, the authors of [15], as well as [16] have applied some of these methods. This study is aimed at analyzing the most appropriate thresholding method for each of the considered CD indices, so that statistical parameters corresponding to the change/no_change categories are properly derived and introduced in a decision rule process.

An additional important point to be considered in CD, and particularly for very high resolution images, is the need to optimize the process by using different types of indicators or ancillary information derived from the same images or other external sources or sensors. Le Hégarat-Mascle *et al.* [12] suggest CD methodologies based on this approach, which main idea was based on the so called ‘multisource classification’ analysis [17–19]. Since then, several authors have applied these approaches to CD processes, from different methodological perspectives. These range from Bayesian methods [4,20], with demonstrated effectiveness and performance according to the results reported in such references when the information is two-dimensional, *i.e.*, change/no-change, up to methods based on the evidential theory of Dempster-Shafer [12,20], Fuzzy Logic [20,21], Level-Set [22] or decision tree methods [23]. A common particularity in these studies is their supervised nature, as the analyst must establish the rules that best fit to a certain algorithm and dataset.

Thus, in this study, a multisource CD methodology based on different change indices, which might be implemented in a non-supervised fashion, is accomplished. This makes the main contribution of this paper. Likewise, an essential part of this study consists in assessing the influence of a particular index during the decision process. For this purpose, thresholding operations are also applied, so that the statistical parameters of the change/no_change categories for each index can be estimated conveniently. Then, the distinct change detection indices and related parameters are integrated into an informational fusion process, and a unique change detection document is derived. Finally, it is also shown that the addressed methodology is applicable and extensible to datasets with different levels of spatial resolution.

This paper is organized as follows: in Section 2 the geographical area and the different multisensor datasets are described. Then, a brief introduction on change detection indices is given. The selected thresholding and informational fusion methods are also presented there. Then, the results reached at each stage of this research are presented and discussed (Section 3). Finally, some conclusions are drawn from the suggested methodology and achieved results.

## 2. Data and Methods

This section describes the data sources used for CD analysis and the main algorithms and methods on which this research is based. First, a set of change detection indices is introduced. Then, the automated thresholding methods used in this work are discussed. Finally, the information fusion theory that allows deriving a change detection map from the previous generated features is explained.

### 2.1. Study Area and Information Sources

**Figure 1.**
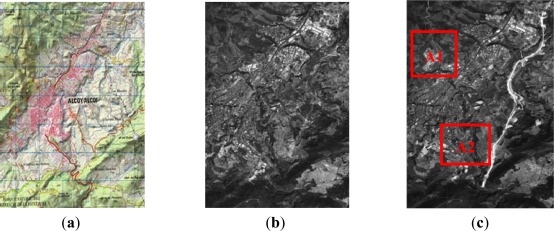
Study Area, (**a**) 1/50000 Official Cartography (^©^IGN-SPAIN, 2005); (**b**) SPOT5 PAN 2005 image (^©^SPOT Image); (**c**) SPOT5 PAN 2008 image (^©^SPOT Image).

The working area used for this study is located on the Eastern part of the Iberian Peninsula, centered on geographic coordinates {φ = 38°42′05″N, λ = 0°28′37″W}, and with an extension of 4.5 × 6.6 km^2^ (Figure 1). This area is constituted of an urban nucleus surrounded by a large rural area. For this analysis, and depending on the data type and its spatial resolution, either the whole area will be taken into account, or it will be limited to certain sub-areas Figure 1(c). This choice is mainly due to the fact that during the years 2005 and 2008, this area has suffered important landcover changes caused by aggressive urban and infrastructure development policies, so it is of special interest for change detection analysis.

For this purpose, separate datasets registered by different optical sensors have been arranged into a 3-level multiresolution scheme according to their spatial and spectral resolutions, the first and the third levels being respectively the lowest and the highest spatial resolution datasets. The first level is formed of two multispectral (XS) SPOT 5 images, with four spectral bands: two visible (G-R) and two infrared (NIR-and MIR) bands. For this work, only the three first bands with the same spatial resolution (10 m) have been used. Similarly, two other images corresponding to the panchromatic mode (PAN) of this system are available. These images have 2.5 m spatial resolution and correspond in this work scheme to the second level. Both modes (PAN-XS) have been acquired simultaneously on 14-08-2005 and 10-08-2008. Their reference in the SPOT image grid system is 272/41 (K/J). In turn, level 3 is made up of 1 m spatial resolution aerial photographs, which have been acquired on October 2005 and September 2007. The corresponding orthoimages have been derived by means of photogrammetric procedures. It must be mentioned that the data corresponding to 2005, have been acquired by means of a ZEISS RMK analogue photogrammetric camera (f:153.992 mm). These aerial photographs have been converted into a digital format through an analogue/digital scanning process. Whereas, the 2007 aerial images have been acquired by means of an ULTRACAM X digital multispectral camera (f:100.5 mm). In this case, only the three visible bands (RGB bands) were available, as they are the unique provided by the supplier. These bands also have a high radiometric resolution of 11 bits, while all remaining datasets bands have 8 bits radiometric resolution.

As mentioned above, due to the high spatial resolution of the images corresponding to levels 2 and 3, it has been decided to work with reduced areas, which are designated as areas A1 and A2 in Figure 1(c). The analysis over the reduced areas does not imply a limit for the proposed approach, neither from the point of view of computational time nor resolution, they are exclusively selected because of the relevant changes they display.

In order to ensure the best geometric fidelity and minimize false alarms during the CD process, it has been verified that all the datasets are geometrically consistent and present disparities at least below their spatial resolution, equivalent to a RMS under 1 pixel. For this purpose, the images were co-registered using the tools provided by a commercial software package [24]. At a first stage, each pair of images to be registered are overlapped and compared visually. Next, a set of at least 30 homologue points covering the full image are automatically gathered for each dataset. The registration is validated when an RMS below 0.5 pixels is achieved. We have verified that for all tested images a second order polynomial adjustment is considered appropriated. All these data have been geodetically referenced to the ERTS89 system with UTM coordinates (Zone 30). Moreover, as aerial images and satellite images are affected by different atmospheric and lighting conditions at each acquisition date, these datasets must be radiometrically corrected or normalized. For this purpose, the approach specified in [8] and applied in [25] has been used in this work. This normalization process, referred to as empirical temporal spectral normalization technique in [25], is based on the linear adjustment of two images in terms of their statistical means and standard deviations.

### 2.2. Methods

According to [26], change detection may be defined, from two coregistered images *I* and *J* acquired on two different dates *t_I_* and *t_J_*, as the production of a map representing the changes occurred on a scene between *t_I_* and *t_J_*. Thus, the resulted document is a binary map with only two classes: change and no-change. This process requires certain operating phases. First a change detection index must be generated. Then, this index is used to characterize the corresponding values for change and no-change categories. This process may be approached by means of a binary thresholding operation. Thus, the extracted information can be inserted into a decision rule procedure, so that those categories are better defined. This section reports a short description on change detection indices and related questions, as well as with the thresholding and multisource analysis algorithms that will be used in this work.

#### 2.2.1. Change Detection Indices

Generally, change detection indices are based on radiometric measurements. The most common are image algebraic operations as the difference or ratio operations [27]. In the case of panchromatic images, the difference image operation is a widely used method, and consists on a simple and straightforward arithmetic difference between the digital values of the two images obtained on different dates (*t_I_* and *t_J_*), the results are easy to interpret as well. Other authors [28] also suggest using the mean difference, aimed to minimize the noise present on the images. An additional widespread method consists on the calculation of the ratio index, which divides band by band the images corresponding to dates *t_I_* and *t_J_*. It has the advantage of reducing the impact induced by some environmental effects such as the solar angle, shadows and terrain topography. It is expressed by:

(1) $$I_{R}\;\left(u,v\right)=\frac{t_{J}\;\left(u,v\right)}{t_{I}\;\left(u,v\right)}$$

In this case, it is preferable to apply a modified version, which uses the ratio of local means corresponding to cells (*u,v*). Each cell (*u*,*v*) identifies the same spatial location once the images *t_I_* and *t_J_* have been co-registered. Its expression is given by Inglada *et al.* [28]:

(2) $$I_{R}\;\left(u,v\right)=1-\text{min}\left(\frac{{\bar{t}}_{J}\;\left(u,v\right)}{{\bar{t}}_{I}\;\left(u,v\right)},\;\frac{{\bar{t}}_{I}\;\left(u,v\right)}{{\bar{t}}_{J}\;\left(u,v\right)}\right)$$

This index has also the advantage of representing jointly the changes occurred in both directions (from-to) depending on the considered date. For multispectral images, a frequently used index is the so-called Change Vector Analysis (CVA). This method generates two outputs: the spectral change vector describing the direction and magnitude of change between images, and the magnitude of total change per pixel which is determined through the calculation of the Euclidean distance between the end points across an *n-dimensional* change space. The main advantage of the CVA method as change detection operator is that any number of spectral bands can be processed, thus generating a single parameter that represents the degree of change between two sets of similar images. Likewise, in order to reduce the spectral dimensionality of a multispectral dataset, it is possible to derive spectral indices as the NDVI (Normalized Difference Vegetation Index), which is sensitive to the surface vegetation content and in turn can lead to a reduction of the computational cost. Thus, image algebra can also be applied to these new derived products. Similarly, it is also suggested to average locally the initial cell values of the *t_I_* and *t_J_* involved images datasets.

Recently, change detection indices based on the so-called similarity measures have also been applied. Initially, these algorithms were used as image registration techniques aimed at identifying homologue points in two images based on their statistical properties. In this sense, [26,27] have applied these methods for change detection [27] suggests that these methods are of particular interest when working with multisensor information, as it is the case with optical and Synthetic Aperture Radar (SAR) images, which is justified by the polarimetric scattering information existing in SAR images. Nevertheless, in this study, all datasets are radiometrically dependent, so these methods will not be taken into account. Instead, due the high degree of spatial information contained in the images of level 3, a different approach has been followed in order to take advantage of this singularity. In this work, this particular issue is addressed by means of the so-called textural measurements. These are computed on a set of adjacent pixels according to some arrangements and related statistical variables, that describe properties such as variability, homogeneity, contrast, mean value, *etc.* This allows deriving new images or textural features that may be used independently or combined with other spectral bands. For this purpose, the well-known textural algorithms of [29], based on the co-occurrence matrix, may be applied. This matrix indicates the number of times a certain cell value appears next to each other. The higher the values in its principal diagonal are, the more homogeneous the texture is. On the contrary, the more scattered the values outside the diagonal are, the more heterogeneous the texture is. In [30,31] it has been shown that these measurements are of great interest when working with very high resolution images, such as those supplied by the IKONOS sensor. However, for specific land covers or landscape scenarios, these algorithms require also appropriate parameters, as a correct window size, and identifying the most convenient textural feature as well. In this study, this approach has also proven to be particularly useful for the highest spatial resolution dataset, where textural features have been derived for each spectral band in the respective images. In this case, the best results have been reached by means of the homogeneity operator with an 11 × 11 window size, as areas with less texture or low entropy but with different radiometric values are observed to be better discriminated. Once all bands have been transformed, the CVA method is applied to these new features or components.

#### 2.2.2. Thresholding Methods

The essential aim of a change detection index is to assist the identification of changes contained on a pair of images of different dates. Basically, this work may be achieved by applying a decision threshold to the histogram of a specific index. Usually, when observing the indices associated to the difference image (modulus), changes values are found on the right of the histogram, while no_changes are located to the left. According to [20], selecting a decision threshold value has an extreme relevance, as the accuracy of the final result (change detection document) will depend mainly on this selection. However, this task is not a simple problem. Some authors [7,32] choose to apply manual threshold selection techniques. On the other hand, in [3,4] the authors decided to implement *ad-hoc* automated procedures. In contrast to these methodologies, [15,16] introduce some well-defined automatic thresholding procedures such as the Otsu or Kittler and Illingworth algorithms. At this stage, it must be pointed out, that all these works are based on the unique index under study, which in turn has been derived from the same image analyzed. In this section, several automatic thresholding procedures are analyzed, in order to apply them further to some change detection indices derived from the image datasets of this study. The thresholding methods considered in this work operate with an image where every cell is associated to a discrete variable *g*, with *g* ∈ [0.255]. These algorithms intend to establish in this interval a threshold value *T* in order to discriminate between change and no_change values. This is expressed as:

(3) No_Change:0≤g≤T and Change:T+1≤g≤G

The sets of cells classified as change (C) or no_change (NC) are statistically distributed, and may be represented by a cumulative probability (*P*) function derived from the normalized frequencies *p(g)* of the cell values. The statistical parameters that define the distribution of the cells values contained in each class, along with their corresponding probability values, allow defining other parameters, as it is the case for entropy, which are useful to establish the optimal threshold value *T*. This value determines the characterization of these two classes, and thus the parameters that define it. For this reason, the automatic thresholding methods have an inherent iterative nature [33]. The first and second-order moments (*μ* and *σ^2^* respectively) calculated from these sets of values are given by:

(4) $$\mathrm{No Change}\left\{ \begin{array}{l} \mu_{\mathrm{NC}}\;\left(T\right)=\sum_{g=0}^{T}g\cdot p\left(g\right)\\ \sigma_{C}^{2}\;\left(T\right)=\sum_{g=0}^{T}\left(g-\mu_{\mathrm{NC}}\;\left(T\right)\right)\cdot p\left(g\right) \end{array}\right.\mathrm{Change}\left\{ \begin{array}{l} \mu_{C}\;\left(T\right)=\sum_{g=T+1}^{G}g\cdot p\left(g\right)\\ \sigma_{C}^{2}\;\left(T\right)=\sum_{g=T+1}^{G}\left(g-\mu_{C}\;\left(T\right)\right)\cdot p\left(g\right) \end{array}\right.$$

In addition to standard thresholding algorithms, such as the so-called iterative and clustering algorithms, other methods have also been analyzed in this work. This is the case, of those based on entropy. Regarding the first group, the Ridler and Calvard method is a common iterative algorithm, which is initialized by means of a threshold value, estimated from the mean value of the histogram entries. The process is iterated from a first threshold *T_n_*, which is calculated from some initial means values. Then, each new threshold *T_n+1_* is computed from the means derived at the previous iteration. The process converges as the tolerance becomes less than or equal to a certain specified convergence value. A more detailed description of this method can be found in [33].

For thresholding methods referred to as cluster analysis, the Otsu method is also a widespread algorithm, and is aimed at establishing two groups or clusters [34]. This method has been selected for this work, as it has already been proved that it delivers satisfactory results [35]. The distribution of the digital value is modeled as the combination of two Gaussian distributions, which are represented as foreground and background values. In this study, these values are respectively represented by the change and no-change categories. The optimal threshold value is derived by minimizing the weighted sum of within class variances. Being *P(T)* the threshold cumulative probability function, the optimal threshold is given by:

(5) $$T_{\mathrm{opt}}=\text{arg max}\left[P\left(T\right)\cdot \left(1-P\left(T\right)\right)\cdot {\left(\mu_{C}\;\left(T_{n}\right)-\mu_{NC}\;\left(T_{n}\right)\right)}^{2}\right]$$

The third group of thresholding methods is concerned with entropy-based algorithms. For a given image, a threshold value *T* is established from the entropy *(H)* of the image values distribution. However, different approaches exist to determine the threshold value based on the entropy. In image processing, entropy is usually associated to a measure of the cells values heterogeneity within a local image window or the whole image. The Shannon entropy *H(T)* is a common measure, and might be applied for change/no-change classes according to:

(6) $$H_{\mathrm{NC}}\;\left(T\right)=-\sum_{g=0}^{T}p_{\mathrm{NC}}\;\left(g\right)\cdot \text{log}\;\;p_{\mathrm{NC}}\;\left(g\right)\;\text{and}\;H_{C}\;\left(T\right)=-\sum_{g=T+1}^{G}p_{C}\;\left(g\right)\cdot \text{log}\;p_{C}\;\left(g\right)$$

The entropy corresponding to the overall image is defined as *H(T) = H_C_(t) + H_NC_(T)*. In this study five methods based on this principle have been considered. The first reviewed entropic procedure is the Kapur method, where the set of cells classified as change and no_change are regarded as two different distributions. The optimal threshold is established when the sum of the entropies corresponding to change/no_change is maximal (Equation (7)):

(7) $$H_{\mathrm{NC}}\;\left(T\right)=-\sum_{g=0}^{T}\frac{p\left(g\right)}{P\left(T\right)}\cdot \frac{p\left(g\right)}{P\left(T\right)}\;\text{and}\;H_{C}\;\left(T\right)=-\sum_{g=T+1}^{G}\frac{p\left(g\right)}{P\left(T\right)}\cdot \frac{p\left(g\right)}{P\left(T\right)}$$

And the optimal threshold is given when:

(8) $$T_{\mathrm{opt}}=\text{arg max}\left[H_{\mathrm{NC}}\;\left(T\right)+H_{C}\;\left(T\right)\right]$$

A more detailed description of this thresholding method may be found in [36]. The second method corresponds to the Li algorithm [37], which suggests minimizing the mutual entropy between the initial and the binary image (thresholded image). This is achieved by a distance measure based on the minimum cross entropy. By applying such theoretical distance, the optimal threshold is formulated as:

(9) $$T_{\mathrm{opt}}=\text{arg min}\left[\eta \left(T\right)\right]=\text{arg min}\left[-\mu_{\mathrm{NC}}\;\text{log}\left(\frac{P_{\mathrm{NC}}}{\mu_{\mathrm{NC}}}\right)-\mu_{C}\;\text{log}\left(\frac{P_{C}}{\mu_{C}}\right)\right]$$

where *η(T)* is a criterion function. The calculation of the optimal threshold involves the evaluation of this function for all possible threshold values [38].

The third considered method is the Shanbhag procedure [39], which is based on the fuzzy logic theory, and takes into account a fuzzy membership coefficient that indicates the degree of membership of every cell in each of the change/no_change categories. The higher the distance of the cell value to the threshold, the greater is the potential of belonging to a given category. On the contrary, the closest it is, the greater the uncertainty. Thus, for any cell value *i* higher or less than a given threshold *T*, the degree of membership (*s*) for each class is defined by the following expression:

(10) $$\begin{array}{l} s_{\mathrm{NC}}\;\left(T-i\right)=0,5+\frac{p\left(T\right)+p\left(T-1-i\right)+p\left(T-i\right)}{2\cdot P\left(T\right)}\\ s_{C}\;\left(T+i\right)=0,5+\frac{p\left(T+1\right)+p\left(T-1+i\right)+p\left(T+i\right)}{2\cdot \left(1-P\left(T\right)\right)} \end{array}$$

The assigned threshold value should have the maximum uncertainty, which is verified when *S_C_ (T)* = *S_NC_ (T)* = *0.5* [14], and the optimum threshold is found for the *T* value minimizing the sum of the fuzzy entropies:

(11) $$T_{\mathrm{opt}}=\text{arg min}\left|H_{f}\;\left(T\right)-H_{b}\left(T\right)\right|$$

where the entropies of Equation (11) are respectively defined as:

(12) $$\begin{array}{l} H_{\mathrm{NC}}\;\left(T\right)=-\sum_{g=T+1}^{G}\frac{p\left(g\right)}{P\left(T\right)}\cdot \text{log}\left(s_{\mathrm{NC}}\;\left(g\right)\right)\\ H_{C}\;\left(T\right)=-\sum_{g=T+1}^{G}\frac{p\left(g\right)}{1-P\left(T\right)}\cdot \text{log}\left(s_{C}\;\left(g\right)\right) \end{array}$$

The next thresholding procedure considered in this study, the Renyi-Sahoo method [40], is similar to the one proposed by [36], and is also based on Renyi’s entropy, *i.e.*, on the probability distributions of the change/no_change classes, and maximizes of the sum of the different entropies, as well as the entropic correlation (*C*(*T*)), which is also defined for the change (*C_C_*(*T*)) and no_change (*C_NC_ (T)*) categories, and depends on the threshold *T*. A parameter ρ must be taken into account to define the *a priori* entropy order. Then the different threshold values are combined in order to derive the optimal value:

(13) $$\mathrm{TC}\left(T\right)=C_{C}\;\left(T\right)+C_{\mathrm{NC}}\;\left(T\right)=-\text{log}\left(\sum_{g=0}^{T}\left(\frac{p\left(g\right)}{p\left(T\right)}\right)\right)-\text{log}\left(\sum_{g=T+1}^{G}\left(\frac{p\left(g\right)}{1-p\left(T\right)}\right)\right)$$

The optimal threshold is reached when:

(14) $$T_{\mathrm{opt}}=\text{arg max}\left\{\mathrm{TC}\left(T\right)\right\}$$

Finally, the last considered method is the Yen method [41] which is a particular case of the Renyi-Sahoo method where a certain value ρ is established as a constant value, usually ρ = 2. Equation (14) holds also for the optimal threshold in the Yen algorithm.

The thresholded images represent the separation between change/no_change categories, which in turn are designated as binary values (0,1). In Section 3, as a first approximation, the selection of the best thresholding result for a given change detection index, will be carried out by means of a verification process. This performance assessment is addressed by means of a quality analysis reported by means of values extracted from the traditional confusion matrices, which in turn will be constructed using check areas, that allow determine the number of achievements and failures for both categories. Thereby, quality measures such as overall and individual accuracies as well as producer’s and user’s accuracies can be drawn from these tables. Regarding the two last accuracy measures, they are important meaningful estimates as they respectively refer to the probability that a certain category (ground truth) is classified as such, and to the probability that a cell value labeled as a certain category in the classified (binarized) document is really this class. Thus, in order to confirm the reliability of the thresholding results, these two measures will be also reported for the best outcomes.

#### 2.2.3. Informational Fusion Methods

In the framework of image classification, and with the aim of improving the end result of such a process, several authors [17–19,42] have suggested methods referred to as multisource data analysis so that it is possible to benefit of the thematic information derived from images acquired by different sensors. According to [43], the integration or fusion of redundant information supplied by different sensors, allows reducing the overall uncertainty and thus the accuracy of the observed features is improved, taking also advantage of the complementary information that may have been observed and acquired by these instruments. These concepts have also been retrieved by [5] and applied in the context of image change detection. Thus, instead of using the multitemporal information supplied by a single sensor, they make use of the different and supplementary information provided by optical and microwave instruments in order to exploit the synergies that might arise from the combinations of the different informational sources.

Meanwhile, different analysis issues have been undertaken, as the fuzzy theory, the evidential reasoning of Dempster-Shafer, the neuronal networks and the Bayesian theory amongst others. Whereas, it is not obvious to decide which of these methodologies is the most appropriate, in this case, the Bayesian theory appears to be most convenient, due to the possibility of adapting it to a non supervised scenario. Moreover, Bayesian estimation provides a solid foundation for merging multisource information so that it may be combined in accordance to the rules of probability theory.

In contrast to other studies related to multisource CD, this work suggests replacing the different informational sources by a specific change detection index, so that every index might have a particular contribution, and can be further assessed through a quality control. Thus, in this case, a set of observed *n* number of variables corresponding to *X_s_* indices will be available, with *s* ∈ {*1*, *n*}. Then, the objective consists in assigning each value within each of these variables to one of the change/no_change categories designed as *ω_j_*. For this purpose, the a priori class information should necessarily be accessible. The method suggested in this work for deriving the a priori probabilities takes into account the best thresholded result for each index, so that each a priori estimate can be computed from the discriminated change/no_changed categories in the thresholded image. According to Bayes theory, the relations between the observed measures in each index and the a priori probabilities will be represented by the membership function *F_j_(x)*, as:

(15) $$F_{j}\;\left(X\right)=p\left(\omega_{j}/X_{1},...,X_{n}\right)=\frac{p\left(X_{1},...,X_{n}/\omega_{j}\right)\cdot p\left(\omega_{j}\right)}{p\left(X_{1},...,X_{n}\right)}$$

On the other hand, [5,44], assume that each informational source (s), by its nature, is affected by a certain reliability factor $\left(\lambda_{s},\sum_{s=1}^{n}\lambda_{s}=1\right)$. Thus Equation (15) may be rewritten as:

(16) $$F_{j}\;\left(X\right)=p\left(\omega_{j}/X_{1},...,X_{n}\right)=\sum_{s=1}^{n}\lambda_{s}\cdot \frac{p\left(X_{1},...,X_{n}/\omega_{j}\right)\cdot p\left(\omega_{j}/\varsigma_{i}\right)}{p\left(X_{1},...,X_{n}/\varsigma_{j}\right)}$$

where *ζ*_i_ is introduced, so that the probabilistic group will depend on the statistical parameters (*μ*, *σ*, *p(ωi)*), which are in turn derived from a defined index as a consequence of the corresponding thresholding process. Consequently, for a set of *n* sources or CD indices *X = [X_1_*, …, *X_n_]*, a value will be classified as one of these two categories in accordance to the maximum likelihood rule:

(17) $$F=\underset{j}{\text{max}}\left[F_{j}\right]$$

As suggested by [45], a different approach of addressing this question is assuming the independence between informational sources, which is judged to be acceptable in this case, given the nature of the operation applied to each change index, the computation of an index is not affected by the computation of other indices. Indeed, values of an NDVI difference change detection (CDI) index does not exhibit the same properties than a CVA CDI and do not hold any relationship, even if both CDI are computed from the same dataset, hence the independence. In these cases, [19] suggest the following membership function for the multisource model:

(18) Fj (X)=p(ωj)⋅∏i=1n{p(ωj/Xi)p(ωj)}λi

and may be rewritten logarithmically as:

(19) log(Fj (X))=log(p(ωj))+∑i=1nαi log{p(ωj/Xi)p(ωj)}

On the other hand, this same concept can be applied in accordance to Bordley’s formulation [46], which considers that the redundant information contained in a set of n sources may be fused using the Odd function (*O*) and the likelihood ratio (*L*), both derived from the Bayes’ theory:

(20) $$L\left(X_{i}/\omega_{j}\right)=\frac{p\left(X_{i}/\omega_{j}\right)}{p\left(X_{i}/{\bar{\omega }}_{j}\right)}\;\mathrm{and}\;O\left(\omega_{j}\right)=\frac{p\left(\omega_{j}\right)}{p\left({\bar{\omega }}_{j}\right)}$$

where *O*(*ω_i_*) are the prior odds and *ω̄_i_* the complementary of class *ω_i_*. Hence, as a consequence of the assumption of independence, the posterior odds, given the different informational sources, are identified by the expression:

(21) $$O\left(\omega_{j}/X_{1},...,X_{n}\right)=O\left(\omega_{j}\right)\prod_{i=1}^{n}L\left(X_{i}/\omega_{j}\right)$$

and the *a posteriori* probabilities are related to the posterior odds through:

(22) $$F_{j}\left(X\right)=p\left(\omega_{j}/X_{1},...,X_{n}\right)=\frac{O\left(\omega_{j}/X_{1},...,X_{n}\right)}{1+O\left(\omega_{j}/X_{1},...,X_{n}\right)}$$

Then, Equation (22) will define the membership function for one of the two change/no_change classes. The latter and Equation (16), are the models that are applied and assessed in the next section. It can also be observed that Equation (21) does not require specifying any reliability factor for each separate source. In addition, the thresholded indices might help to approximate for each change detection index either, the *a priori* probabilities of each category, as well as the statistical parameters necessary to assess their corresponding density probability functions.

**Figure 2.**
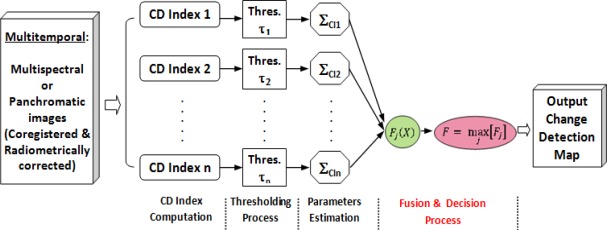
Methodological framework.

The different phases of the proposed methodology are then gathered in the flow-diagram depicted in Figure 2.

First, the registered and radiometrically normalized datasets specified in Section 2.1 are used as initial input variables. Then, change detection (CD) indices are generated. Although it would be possible and desirable to use more than two CD indices, in this work, only two indices have been used at each resolution level. The rationale of this choice is simply due to the need of measuring efficiently the variation of the weighting factors along the integration process, as this allows assessing correctly the contribution of each index. Next, thresholding processes are carried out and their results are analyzed. Finally, the multisource integration processes as well as the corresponding quality analysis are addressed. Likewise, for assessing the quality of these two informational fusion methods, the method based on confusion matrices and their related accuracy estimates will be applied as suggested in Section 2.2.1.

## 3. Results and Discussion

In this section, the results obtained from the above-mentioned processes and illustrated by means of Figure 2 are presented and discussed.

### 3.1. Change Detection Indices

Change detection index processing has been carried out in a different way depending on the dataset considered and its resolution level, as it has already been pointed out in Section 2.1. However, it is very important to emphasize that, as a result of the distinct spectral properties of the involved datasets, different indices have been derived at each resolution level. Although, the choice of a particular index may be scene dependent, this work is mainly focused on testing different thresholding procedures as well as on assessing the mentioned informational fusion procedures. For this reason, this study is limited to the same geographical area.

**Figure 3.**
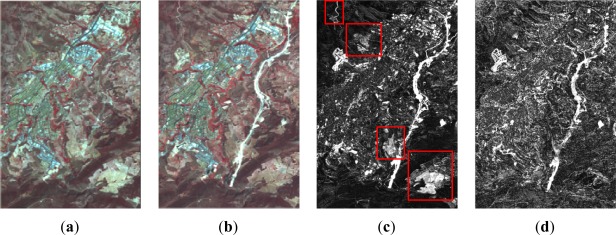
Level 1 processing, SPOT5 XS (**a**) 2005 normalized image; (**b**) 2008 image; (**c**) CD CVA index; (**d**) CD NDVI difference index.

Thus, for the first dataset (resolution level 1) the CVA method and the NDVI difference index have been applied. For the first change detection index bands 1 (green), 2 (red) and 3 (near infrared) have been used. Whereas for the second change detection index only bands 2 and 3 are required. Figure 3 shows the normalized (earlier date) and the reference (later date) images, as well as the derived change detection indices for level 1.

As it is appreciated, in addition to the similarities exhibited in both cases, some differences can also be recognized and are shown in Figure 3(c). Moreover, some changes are better highlighted depending on the given index. This is due to the fact that the CVA method is based essentially on the chromatic differences between the two images. Whereas, the NDVI difference is not influenced by radiometric effects, so it may be useful to provide complementary information in the cases of vegetation changes. Nonetheless, in some situations it is very difficult to decide which are due to real changes or simply produced by lighting conditions, as it might happen in the lower right corner of these images Figure 3(c).

**Figure 4.**
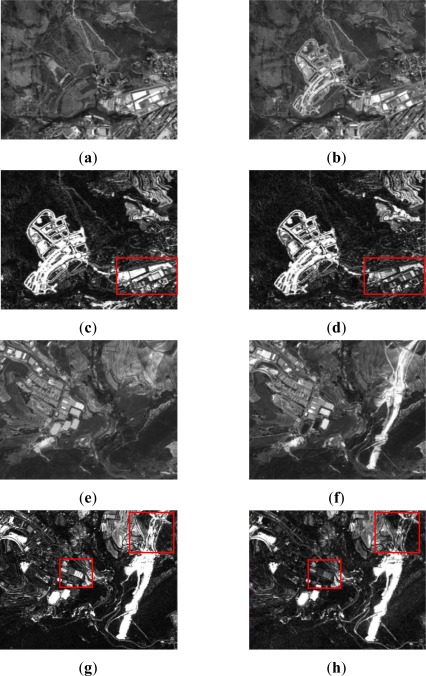
Level 2 processing, SPOT5 PAN (**a**) area A1: 2005 image; (**b**) area A1: 2008 image; (**c**) area A1: CD Difference index; (**d**) area A1: CD Ratio index; (**e**) area A2: 2005 image; (**f**) area A2: 2008 image; (**g**) area A2: CD Difference index; (**h**) area A2: CD Ratio Index.

For resolution level 2, where panchromatic images are used, the simple difference and the ratio are two appropriate change detection indices for this type of data. As it has been mentioned before, for this level, the study has been restricted to two working areas, which are shown along with their corresponding change detection indices in Figure 4. For this level, after a preliminary visual survey, almost no dissimilarities are observed between the difference and ratio CD indices. However, as it is pointed out in Figure 4(c,d) (area A1), high grey level variations are appreciated, which indeed represent real changes between the two dates Figure 1(a,b).

In area A2 (Figure 4(g,h)) some differences between the two indices are still recognized, but they are not as apparent as in the previous case. These preliminary results enforce the idea that different indices derived from the same image may also contain complementary information. This means that a particular change detection index can highlight some information which is not shown by another index, and vice versa, as shown in Figure 4(c,d,g,h). Thereby, change detection indices complement themselves.

**Figure 5.**
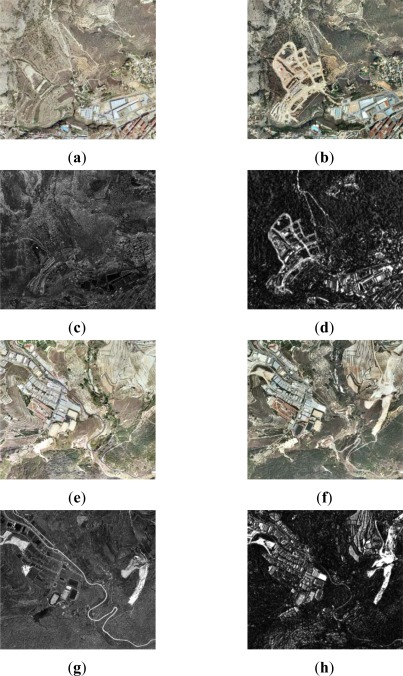
Level 3 processing, aerial images (**a**) area A1: 2005 Orthoimage; (**b**) area A1: 2007 Orthoimage; (**c**) area A1: CD CVA index; (**d**) area A1: CD Texture-Homogeneity CVA index; (**e**) area A2: 2005 Orthoimage 2005; (**f**) area A2: 2007 Orthoimage; (**g**) area A2: CD CVA index; (**h**) area A2: CD Texture-Homogeneity CVA index.

For level 3, the corresponding processes have been applied to the same areas. Although, these data are also multispectral images, the near infrared band is not available, so it has been impossible to derive the CD NDVI difference index. Subsequently, the CVA method appears to be most suitable for this purpose. Moreover, as it has been anticipated in Section 2.2.1, an additional CVA index has been computed using textural features based on the homogeneity operator. Figure 5 shows the results of the CVA method applied to the original dataset of level 3, as well as to their corresponding textural features. At this stage, the differences between the derived CD indices are properly distinguished. Although, in both cases the same method is applied, the most notable differences are observed when the textural measures are introduced Figure 5(d,h). Finally, and for convenience, all indices based on the difference index, have all been transformed into positive values by applying the absolute value function.

### 3.2. Thresholding Processes

The results achieved along the thresholding processes are summarized in Tables 1–3. Each resolution level dataset is analyzed separately, and the applied algorithms are arranged according to their particular principle, as described in Section 2.2.2. For each index, the best thresholding method is designated with bold characters.

For level 1 and the CD CVA index (Table 1), the best solution has been reached applying the Li method, with 86.4% overall accuracy. Likewise, individual accuracies for the two categories (change and no-change) are higher than 85%, which indicates a good performance of this type of algorithm for this change detection index. This improvement is also reported by the producer’s (85.5% and 87.4%) and user’s accuracies (85.5% and 87.3%) derived from the confusion matrices, for change/no_change categories respectively, which also ensure the goodness of this thresholding method for discriminating these two classes in the referred CD index. References about the correct outcomes of the Li procedure have not been reported in the literature, except those given by the own authors [37,38].

The satisfactory results achieved by the CVA index of SPOT5 multispectral images, might certainly be due to the medium-high range of different changes values found by the CVA operator in this study area, which in fact are seen by the algorithm as a high entropy or informational content. As a consequence, this CD index is adequately binarized by this method. Probably, for this type of data, minimum cross-entropy procedures deliver better results than those based uniquely in density and distribution functions, as well as those based on fuzzy entropies.

**Figure 6.**
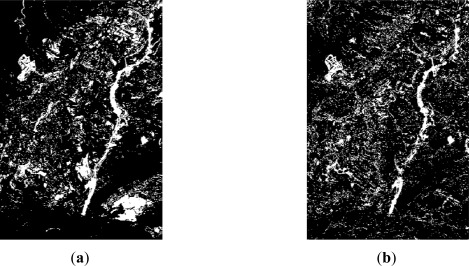
Level 1 thresholded indices: best results (**a**) CVA–Li method (binary image); (**b**) NDVI–Otsu method (binary image).

Always for level 1, in the case of the difference CD index based on NDVI’s, it is found that the information content is comprised between the value range [0,1], which implies that the change/no-change categories values are close to each other or within a short range of values. According to [14,47] the Otsu thresholding method provides satisfactory results when the measures of each category are similar, which is indeed the case for the classes contained in this index. These observations are formulated for the Ridler-Iter procedure [33] as well. While, the best results are achieved by these two methods, the overall accuracy does not reach in any case 80% and the individual accuracies for the change category are also not as expected (66.5–68.5%). Similarly, for change/no-change classes, producer’s (68.5% and 87.4%) and user’s (82.7% and 76.0%) accuracies also decrease, which may indicate that the NDVI CD index might not be suited for this particular geographical area. Nonetheless, the outcomes of these two procedures exhibit better a quality compared to those based on entropic algorithms. The result of the Otsu method is thus selected for further processes, as it shows a better overall accuracy and kappa coefficient. Figure 6 shows the best results obtained for level 1 in agreement with the quality values given previously. For the CVA case, it is confirmed that the radiometric differences tend to be overrated, while in the second case (NDVI difference index) these changes are not detected Figure 3(d). However, in both cases, the thresholded images have been solved adequately with the selected methods (Table 1).

Concerning level 2, the results of the different thresholding methods are presented numerically in Table 2. In this case the operations have been carried out on the two study areas indicated in Figure 1(c), and organized in Figure 4(a,b,e,f).

In Table 2, it is observed that the Li method performs correctly for both indices. Indeed, in comparison with other results, for the difference index, in the case of the two selected study areas, this method delivers the best accuracies. For the overall accuracies, they differ between 85% and 90%, with a good agreement Kappa coefficient. In area A1, for this thresholding method and the difference operator, good producer’s accuracies are reached for the two considered categories (change/no_changed), 85.6% and 84.1%. Regarding user’s accuracy, these values are similar, 85.3% and 84.5. Whereas, in area A2 very good values are obtained in both cases and classes: 87.9% and 92.2% (producer’s acc.–change/no_change), 89.5% and 91.0% (user’s acc.–change/no_change) Moreover, it might also be confirmed, that this entropy-based method solves appropriately situations where a large informational content is present, as it happens in this level, which has 2.5 m of spatial resolution, in comparison to level 1 with only 10 m, although the increase of information may also be responsible of introducing some degree of noise.

As a consequence of the values given in Table 2, the Li method produces good outcomes either for the difference index as for the ratio index. However, in the case of the ratio index, similar results are also observed for the Iter and Otsu methods. These two methods are even slightly more accurate (0.5%) than the Li method. Additionally, for these two methods and area A1, producer’s accuracies are 82.1% (change) and 87.8% (no_change), as well as 87.8% (change) and 82.1% (no_change) for user’s accuracies. Furthermore, for area A2, 74.8% (change) and 94.5% (no_change) producer’s accuracies are reached, while 91.2% (change) and 83.3% (no_change) user’s accuracies are obtained. These values also differ slightly from the Li case. As a consequence of these results, the outcomes of these thresholding methods applied to the ratio CD index are considered acceptable.

**Figure 7.**
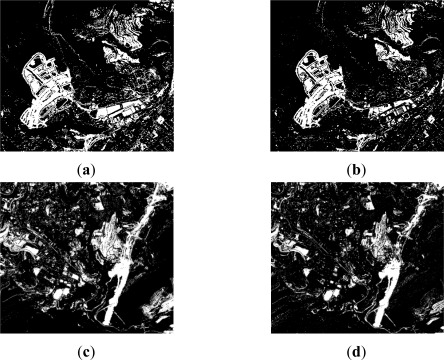
Level 2 thresholded indices: best results (**a**) Area A1: Diff. Index–Li method (binary image); (**b**) Area A1: NDVI–Otsu method (binary image); (**c**) Area A2: Diff. Index–Li method (binary image); (**d**) Area A2: Ratio Index–Otsu method (binary image).

Regarding the variability range of the ratio index values, it appears to be shorter (NV_rat_min_ = 0, NV_rat_max_ = 9) than the values interval of the difference CD index (NV_dif_min_ = 0, NV_dif_max_ = 68). As it has been stated by [14], in these cases, iterative and clustering algorithms, as the Iter and Otsu procedures, perform better than entropic methods, as it happens to a certain extent with the Li method and clearly with the remaining entropy based algorithms. In order to verify this issue, a visual analysis has been carried out, which has definitely confirmed the suitability of the Otsu and Ridley-Iter methods. The final results corresponding to the most appropriate thresholding methods are depicted in Figure 7. Comparing these methods with the change detection indices shown in Figure 4, it may be stated that the applied procedures lead to a reliable representation of the change and no_change areas.

At this second level of resolution, it can also be appreciated that different thresholding methods lead to similar results, as it was the case for level 1. Moreover, for both levels, with different sensor modes, Pancromatic *vs.* Multispectral, and 2.5 *vs.* 10 m spatial resolution respectively, the Li method has yielded the best accuracies for the CD indices with higher variances, and the iterative and clustering methods for the lowest, as it is the case for the NDVI and ratio indices. Thus, this implies that the statistical variance of the index plays an important role when selecting a specific thresholding algorithm.

Finally, for level 3, as it could be expected from the change detection index shown in Figure 5(c,h), some difficulties in the thresholding results have also been encountered. These are originated due to the initial properties (analogue to digital conversion) of the dataset corresponding to the earlier date, which has been explained in Section 2.1.

**Figure 8.**
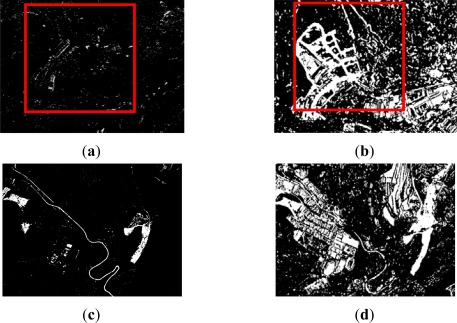
Level 3 thresholded indices: best results (**a**) Area A1: CVA Index-Yen method (binary image); (**b**) Area A1: CVA-Text-Hom.–Li method (binary image); (**c**) Area A2: CVA Index–Yen method (binary image); (**d**) Area A2: CVA-Text-Hom.–Li method (binary image).

Subsequently, the deficiencies identified in the corresponding change detection indices are also transmitted to the thresholding results. As it may be expected, the CVA method delivers poor results (Figure 8(a)). However, for the second analyzed area (Figure 8(c)), it can be appreciated, that the Yen method exhibit correctly certain changes contained in the input data file (CVA index), thus yielding an improvement in the accuracy. This result might be due to the capability for solving the compactness between categories of the Yen method [41].

Change and no_change classes are also observed to be well discriminated in the CD index Figure 5(g). This achievement is closed to the Shanbhag and Reny-Sahoo thresholding results, which are also referred to as entropy based methods. Nevertheless, despite of this improvement, this index will not contribute with a high quantity of information to the fusion process.

On the other hand, the calculation of textural measures and its subsequent processing through another CVA index has provided a solution to this problem. Contextual operations performed by the homogeneity textural operator have corrected the degraded radiometric information in the analogue dataset, which apparently has resulted in a global enhancement, and is in accordance with [12,48], who have pointed out the importance of considering these operators for change detection processes in the case of high spatial resolution images. This solution has provided here a successful alternative for solving problems related with radiometrically deficient datasets.

Thus, the CVA CD index computed with these new features is thresholded adequately by means of iterative (Ridler), clustering (Otsu) and entropic (Li) algorithms. However the latter exhibits a better overall performance, 79% *vs.* 76% (by averaging the overall accuracy of both areas). Producer’s and user’s accuracies exhibit also similar values, so they are not reported here. Moreover, revising the statistics of this CD index, its dynamic range varies from NV_min_ = 0 to NV_max_ = 140, which is a rather large interval denoting an important informational content. Thus, this last result confirms again the usage of the Li method. The thresholding results corresponding to this index (CVA_text_) are depicted graphically in Figure 8(a–d), where the differences with the thresholded CVA CD index derived from the original dataset, are clearly highlighted. An obvious increase of information can be appreciated when textural measures are taken into account.

In this section, several methods belonging to different thresholding families have been assessed for binarizing change detection indices. These methods have been applied to images acquired by specific spaceborne and airborne sensors at different dates. It has been confirmed that the entropy-based methods are appropriate for images with a high degree of information. This appears reasonable when the spatial resolution of the images increases, but also for datasets with high dynamic ranges as it happens with the CVA of the SPOT multispectral dataset. Whereas, for images with change and no_change categories values close to each other, the iterative or clustering methods provide a better alternative as it has been the case for the Ratio or NDVI’s difference CD indices. Although, this statement should also be checked in other geographical environments. Furthermore, as it is denoted by Chan *et al.* [47], the results obtained using the proposed thresholding methods show that each of these algorithms produce a different result and that the proper implementation of a particular thresholding method does not necessarily imply that it is optimal for another index (or not even for a different dataset).

Then, once the best solutions at each resolution level are identified, each thresholded image can be used to extract from its corresponding change detection index the statistical parameters for change and no change categories. This task has been achieved employing these images as mask layers, performing statistical zonal operations based on their binary values. The corresponding result allows providing the probability density functions with the required parameters for the correct evaluation of the CD decision rule process using the multisource analysis suggested in Section 2.2.3. In this work, all categories populations are considered to be normally distributed, so mean values and standard deviations are the values required carrying out the following working stage. Bruzzone and Prieto [4,5] have studied and assumed normal distributions for histograms representing image differences, after different studies this assumption has also been considered, verifying its correctness.

### 3.3. Informational Fusion Analysis

In this section, the summative (Σ) and multiplicative (Π) methods described in Section 2.2.3 are implemented and evaluated. For this purpose, the extracted statistical parameters, for change and no_change categories of each processed CD index, are used. This analysis has been performed for the three considered resolution levels. Furthermore, by means of this multisource methodology, the contribution of each CD index is also examined, as each index is also affected by a reliability or weighting factor (Equation (16)). However, in the case of Equation (22), this possibility is not considered, as the inclusion of different factors in the process will indeed not produce different results. In the extreme cases where λ_s_ = 0, the result will always be null as well.

All results are gathered in tables, where the accuracies reached at each resolution level are represented. For this purpose, the same check areas used in Section 3.2 have been used for building up the corresponding confusion matrices and deriving overall accuracies, as well as other related accuracies reached for each category. True positives and false negatives rates are also reported [49,50]. The best results reached at each resolution level are also represented graphically using rose plots. Moreover, ROC (Receiver-Operating-Characteristics) curves have also been constructed for better interpreting the performance of the experiments carried out for each resolution level.

Table 4 shows the results of the summative and multiplicative multisource processes for level 1. In both cases, the best achieved overall accuracy is higher than 85%. In this table and for the summative process, the outcomes for different index combinations and their corresponding reliability factors are represented. In particular, the contribution of the CVA index is observed to be higher than the NDVI difference index, which improves only a 0.2% for similar weighting factors (λ_CVA_ = 0.5 or λ_CVA_ = 0.6), and where the agreement coefficient (κ = 0.73) is also slightly better. This might also prove that when two indices are taken into account in such a process, the final result is not disturbed and can also benefit from the informational content of the two indices. For the reported combination n° 7 in Table 4, satisfactory producer’s accuracies are reached for the change/no_change categories: 84.5% and 88.5% respectively. Likewise, similar values are also obtained for user’s accuracies and the same categories, 86.6% and 86.7%. This implies a good performance of the change/no_change assignment process by means of this informational fusion procedure. These values do not differ for the remaining combinations with overall accuracies around 86%.

Concerning the multiplicative method, a unique result is reported, as different weighting factors combinations do not produce different results. Also, combinations 1 and 11 (Table 4) are unsuitable, as multiplication by a null factor will produce a result with null values as well. Thus, these cases should also be avoided for the multiplicative method as it is suggested in this paper. These situations are denoted by an x in Tables 4 and 5. For this unique result an 86.1% overall accuracy has been reached, which is close to the best outcomes of the summative method (cases 6 to 11 in Table 4). In this case, analogous remarks can also be drawn regarding producer’s and user’s accuracies, as well as about the assignment process. Producer’s accuracies for these categories are 81.4% and 90.2%, while user’s accuracies are 88.0% and 85.6%, which can be considered quite acceptable.

**Figure 9.**
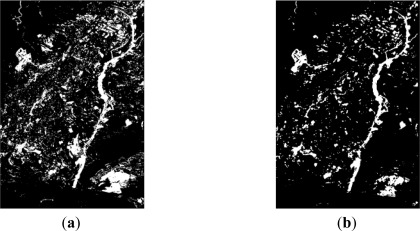
Level 1. Change Detection documents (λ_CVA_ = 0.6, λ_NDVI_ = 0.4) (**a**) Summative method; (**b**) Multiplicative method.

The results reached for both methods, summative and multiplicative, are shown graphically in Figure 9. It is interesting to observe how the multiplicative method helps to reduce, to a certain extent, some degree of noise, while preserving the most representative changed or unchanged areas as detected by the corresponding change detection indices. However, this may lead to no desired consequence, when a specific index underestimates seriously one of the two categories. This problem will be further appreciated in level 3. Nonetheless, for this resolution level and dataset, these two informational fusion procedures appear to be suitable methods for categorizing change and no-change classes. Moreover, the summative process allows also identifying the best CD index or combination of indices.

The results achieved for level 2, where panchromatic data with 2.5 m spatial resolution has been used, show a similar performance for the two multisource methods. It should be noted that for assessing this level, and for the sake of simplicity, the accuracy measures for the two study areas have been averaged, assigning mean values to the quality measures of the summative and multiplicative processes. These results are displayed in Table 5. In this case, the overall accuracy of the implemented summative method improves a 2% in comparison with the best implementation in level 1. An overall accuracy of 88.3% is achieved, which appears to be a satisfactory result. Also producer’s and user’s accuracies are very satisfactory for combinations with this overall accuracy. These results are reported here for the combination n° 7 (Table 5), as it has delivered slightly better results. In the case of the producer’s accuracy 86.4% (change) and 89.9% (no-change) values have attained, while 88.0% (change) and 88.5% (no-change) values have reached for user’s accuracy.

**Figure 10.**
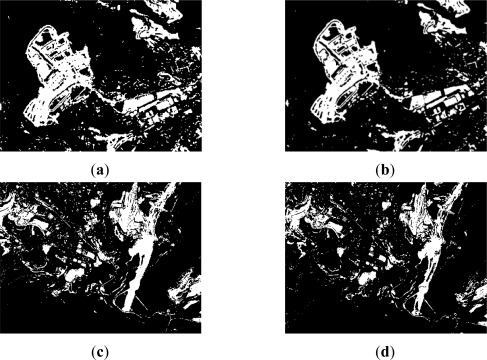
Level 2. Change detection documents (λ_CVA_ = 0.6, λ_NDVI_ = 0.4) (**a**) Summative method-area 1; (**b**) Multiplicative method–area 1; (**c**) Summative method-area 2; (**d**) Multiplicative method-area 2.

Again, the indices behave differently, and the difference CD index shows a higher contribution. However, the ratio CD index does not disturb in any case this process when combined properly with the other index. Furthermore, when different reliability factors (λ_DIFF_ = 0.6, λ_RATIO_ = 0.4) are applied to these indices, the results tend to remain unchanged with the highest accuracy rate. This would imply that while the difference index plays a more relevant role in this process, the second index is also important. Hence, it is suggested to keep both indices, as they might contribute jointly to this end result. The results corresponding to the summative method applied to level 2 are represented graphically in Figure 10(a,c).

Regarding the multiplicative process, the overall accuracy has decreased a 2% compared to the summative informational fusion method. However, this score is still satisfactory with an 86.1% overall accuracy (Table 5). Values for producer’s accuracy are 79.5% (change) and 92.0% (no_change), whereas values for user’s accuracy are 89.5% (change) and 83.9% (no_change). Even though, these accuracies are considered satisfactory. Despite this loss of overall accuracy (2%), the same effect regarding noise is noticed for this level and method Figure 10(b,d). Therefore, it can be stated that the multiplicative algorithm considered in this study is able to supply also suitable results, when the dataset is constituted of reliable indices such as the difference and ratio indices. Nonetheless, again the weights factors do not play any relevant role in this procedure.

At this point, it should be mentioned, that the accuracies measures reached for these two levels have also improved slightly in contrast to those achieved individually by the thresholding techniques specified in Section 3.2. In fact, those results could be regarded as change detection documents. However, this informational fusion implementation allows a better integration of those results, rather than combining directly the thresholded indices.

Thus, regarding the results reached at these two first levels, it is evidenced that summative and multiplicative procedures referred to as multisource methods [17–19,42,43], are able to provide suitable methodological frameworks for integrating different indices derived from a same dataset in order to produce change detection documents. With the first method it is also possible to asses which of the indices provide the most significant informational content. Furthermore, due to the promising results reached with the second method, additional efforts must still be carried on for optimizing this methodology. It is also very important to note that the thresholding operations have supplied a solid base for extracting statistical parameters corresponding to change and no_change categories.

Unfortunately, it is not possible to draw the same observations and comments for level 3. As it has been discussed in the previous section, this case presents certain difficulties caused by the radiometric deficiencies in the first date of the dataset. Even though, this problem was partially overcome deriving textural features, it has not been sufficient to reach acceptable results. For this reason, the achieved values for the quality measures shown on the previous tables are not all given, and only the most representative outcomes are reported for this third level.

**Figure 11.**
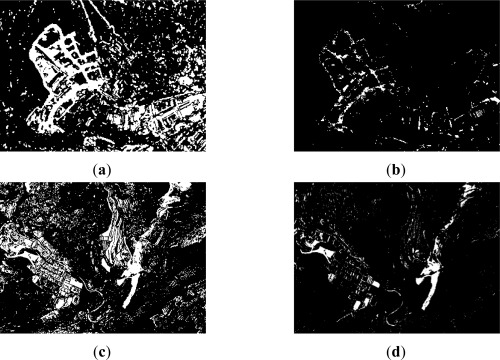
Level 3. Change detection documents (λ_CVA_ = 0.6, λ_NDVI_ = 0.4) (**a**) Summative method–area 1; (**b**) Multiplicative method–area 1; (**c**) Summative method-area 2; (**d**) Multiplicative method-area 2.

The differences between the results accomplished for levels 2 and 3 can be appreciated in Figures 10 and 11, which show clearly the bad performance attained in the third level for both informational fusion procedures. In addition, some degree of false alarms and noise is also observed in level 3 for the summative method Figure 11(a,b).

In this sense, a medium accuracy has been achieved for level 3 with the summative procedure. The best results are obtained when larger weights are assigned to the textural CVA index (λ_CVA_ = 0.3, λ_CVA-T_ = 0.7 and λ_CVA_ = 0.4, λ_CVA-T_ = 0.6), with 79.7% overall accuracy, although similar accuracies are also reached exclusively by this CD index. Regarding producer’s and user’s accuracies, these are not evaluated due to the results achieved at this level.

On the other hand, this value decreases rapidly, when higher weights are assigned to the second index. In these cases the overall accuracy is around 60%, and the remaining quality measures do not exhibit better values. This poor accuracy is also observed for the multiplicative method, which is in fact produced by the absence of information in the CVA index. Therefore, in such cases, this method tends to eliminate the information contained in the other index. This consequence can be clearly noticed in Figure 11(b,d). As a final remark regarding this level of resolution and dataset, it is considered that the accuracies would have been higher with images registered exclusively by digital sensors, as the one used for the second date. Moreover, when dealing with very high spatial resolution datasets, additional ancillary information must also be taken into account, such as Digital Surface Models (DSM) in urban environments, as well as other integration strategies, like those suggested in [12,50]. Despite these poor results, this experiment also demonstrates that this evaluation methodology is able of providing an issue for assessing the contribution of a particular index.

**Figure 12.**
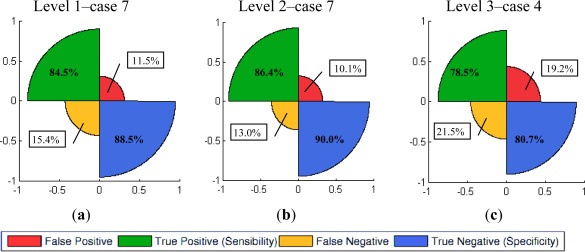
Summative method (Σ) rose plots (**a**) Level 1-case 7 (Table 4); (**b**) Level 2-case 7 (Table 5); (**c**) Level 3.

The last part of this section describes the graphical representation of the quality measures for the three levels of resolution. For the summative method, only the best results (cases 7–l1, 7–l2 and 3–l4) achieved for each level are shown in Figure 12. For this purpose rose plots [51] have been used. These diagrams are constructed from the information contained in confusions matrices, their different sectors indicate the percentages of true and false positives, as well as the corresponding negatives. The high proportions of true positives and negatives *vs.* the lower proportions of false negatives reveal the good performance of this informational fusion method for levels 1 and 2 for the given cases. This tendency changes for level 3, where the sectors corresponding to the false positives and negatives increase noticeably.

**Figure 13.**
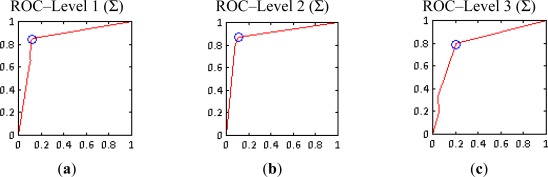
Summative method (Σ) ROC curves (**a**) Level 1; (**b**) Level 2; (**c**) Level 3.

Furthermore, ROC (Receiver Operating Characteristic) plots have been used to represent the performance of the complete experiment carried out at each resolution (Figure 13), where the true positives and false negatives rates for each set of weighting factors combination are plotted in these spaces. The resulting curve and the area enclosed with the horizontal axis denote the reliability of the implementation of the conducted tests.

These curves demonstrate that the better performance corresponds to the second level, where SPOT5 Panchromatic data have been employed Figure 13(b). As the informational fusion procedures are the same for the three cases, this result proves that the panchromatic images are able to deliver better results in comparison with multispectral data, in this case SPOT5 XS. Instead, the curve derived for level 3 shows that the results at this level are less satisfactory, as it was expected. Moreover, blue points in Figure 13 indicate the best rates at each resolution level, which corresponds to the cases highlighted in Tables 4 and 5 for levels 1 and 2.

**Figure 14.**
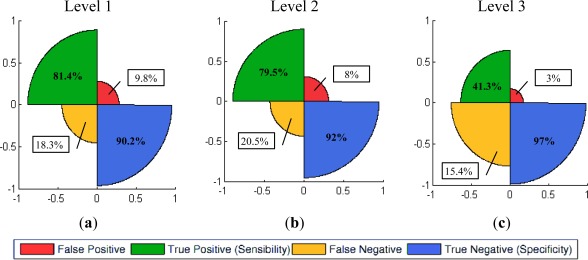
Multiplicative method (Π) rose plots (**a**) Level 1; (**b**) Level 2; (**c**) Level 3.

Similar illustrations have been employed for the multiplicative method. Regarding Rose plots corresponding to levels 1 and 2 (Figure 14(a,b)), a reduction in false positives is observed in benefit of the no-change category, which would imply that an important part of the noise has been assigned to that category, as it has been perceived in Figures 9 and 10.

**Figure 15.**
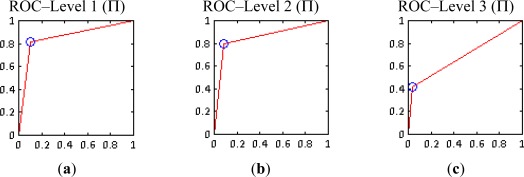
Multiplicative method (Π) ROC curves (**a**) Level 1; (**b**) Level 2; (**c**) Level 3.

Regarding level 3, although a similar trend is also observed, this representation depicts the deficient results reached for this level (Figure 14(c)). Finally, for representing the results of the multiplicative method by means of ROC curves, a unique solution was accessible for each level, so these curves are built up based on these single results (Figure 15).

ROC curves for levels 1 and 2 exhibit a correct performance (Figure 15(a,b)), while the corresponding curve for level 3 (Figure 15(c)) is even worse than the curve depicted in Figure 13(c), which explains the deficient quality of the images shown in Figure 11(b,c). However, in general, these curves must also be interpreted carefully, since they only represent a unique test and do not represent an entire experiment, as it the case for the summative method.

As a consequence of these studies, these multisource analysis methods have shown a great potential for integrating different change indices, as well as for assessing their contribution in a change detection scheme. Moreover, the outcomes reached by means of any of the suggested methods, summative or multiplicative, are a direct consequence of the data quality and the corresponding derived indices, and do not depend on the spatial resolution level. Furthermore, an important amount of noise and/or false alarms has been encountered in the different results, although the data was previously filtered with traditional noise reduction methods. This question, simultaneously with the implementation of additional non-parametric procedures capable of integrating efficiently different change detection indices, must also be addressed in future studies.

## 4. Conclusions

In this work, a set of methods and algorithms have been implemented aimed at obtaining change detection documents using data acquired by airborne or spaceborne optical sensors. Due to the nature of the data provided by these systems, it has been possible to derive several change detection indices for the different multiresolution datasets. This study has been carried out with two change indices at each spatial resolution level, so that the multisource integration algorithm can benefit from the complementary information exhibited by the corresponding indices. As the spatial resolution of the images increases, the use of textural features based on the co-occurrence matrices provides an alternative for enhancing the contextual information and solving some problems associated with radiometric deficiencies, as it has been the case with in this work for a particular dataset.

The automated thresholding algorithms tested in this study confirm their sensitivity and efficiency for differencing between change and no_change categories, and allows estimating precisely the statistical parameters of these classes. For the two first resolution levels, these categories have been determined with at least 85% overall accuracy. Nevertheless, there is no universal thresholding method, but rather each method may be more appropriate for a specific change detection index. For CD indices such as the CVA or the difference index, iterative and clustering methods have produced better results, while for entropy algorithms the Li method has yielded a better performance. Hence, the application of these methods depends on the index dynamic range. Furthermore, these algorithms have proved to assist efficiently change detection procedures regardless of the spatial resolution level of the considered sensors.

The chosen informational fusion algorithms are appropriate for the implemented change detection methodology. Overall accuracies of 86.7% for level 1 and 88.7% for level 2 have been achieved with the summative method. In the multiplicative case, correct results are also reached, 86.1% and 86.2% for level 1 and 2 respectively. Due to the problems encountered in level 3, it has not been possible to reach good accuracies. However, it is expected that with data acquired exclusively by means of digital airborne sensors, the quality of the different derived products (change detection indices) and related results (threshold and change detection documents) may improve considerably. Thus, this study has verified that, while a change detection process may be accomplished only by means of a single change detection index, it is also recommended to consider additional alternatives or change detection indices before adopting a definitive option. The implications of this study are that new issues are still open for optimizing the methods suggested in this work, as well as the significance of always considering more than one index in a change detection scheme. In this work we have assumed that the histograms of differences follow a normal distribution, future studies could be carried out to accurately analyze the form of such distributions. Regarding the optimum weights in the informational fusion procedure, they will be influenced by each image dataset, as well as on their derived change detection indices and the related geographical area.

As a final conclusion, because in image change detection noise presence can be a serious problem, due to the occurrence of false changes, in further studies it should be of interest to apply techniques for noise removal, perhaps as a posterior step to image change detection. Optimization approaches applied in some previous works have been proven with satisfactory results in different environments and conditions. These techniques try to remove noisy pixels mislabelled as change/no-change based on the information supplied by their neighbours. So, a pixel labelled as change could be relabelled as no_change if the majority neighbours have been labelled as belonging to this last category and vice-versa [52].

## Figures and Tables

**Table 1.** Individual and global accuracies obtained for the thresholded indices corresponding to level 1.

**Thresholding Method**	**Class**	**CVA**			**DIFF. NDVI**		

		**Individual Acc. %**	**Overall Acc. %**	**Kappa Coeff.**	**Individual Acc. %**	**Overall Acc. %**	**Kappa Coeff.**
Ridler—Iter	C	68.0	82.2	0.64	66.5	78.0	0.55
	NC	94.6			88.1		
Otsu	C	69.1	82.6	0.64	**68.5**	**78.6**	**0.56**
	NC	94.5			**87.4**		
Kapur	C	44.0	72.7	0.43	1.25	53.8	0.01
	NC	98.0			100		
Li	C	**85.5**	86.4	0.73	83.4	74.5	0.50
	NC	**87.4**			66.6		
Shanbhag	C	3.7	54.9	0.04	36.5	67.5	0.32
	NC	100			94.9		
Renyi-Sahoo	C	44.0	72.7	0.43	2.8	54.5	0.03
	NC	98.0			100		
Yen	C	44.02	72.7	0.43	1.1	53.7	0.01
	NC	98.05			100		

**Table 2.** Individual and global accuracies obtained for the thresholded indices corresponding to level 2, areas A1 & A2.

**Thresholding Method**	**Class**	**DIFFERENCE RATIO**											
		**Individual Acc. %**		**Overall Acc. %**		**Kappa Coeff.**		**Individual Acc. %**		**Overall Acc. %**		**Kappa Coeff**	

		**A1**	**A2**	**A1**	**A2**	**A1**	**A2**	**A1**	**A2**	**A1**	**A2**	**A1**	**A2**
Ridler—Iter	C	63.9	70.8	76.0	86.6	0.52	0.72	**82.1**	**74.8**	**84.8**	**86.1**	**0.70**	**0.71**
	NC	88.9	98.5					**87.7**	**94.5**				
Otsu	C	65.0	71.4	76.4	86.8	0.53	0.72	**82.2**	**74.8**	**84.8**	**86.1**	**0.70**	**0.71**
	NC	88.7	98.4					**17.8**	**94.5**				
Kapur	C	44.2	56.1	67.7	80.8	0.36	0.59	62.4	56.0	78.6	80.5	0.58	0.58
	NC	92.9	99.4					95.9	98.9				
Li	C	**85.6**	**87.9**	**84.9**	**90.4**	**0.70**	**0.80**	**89.9**	**85.3**	**85.9**	**84.2**	**0.72**	**0.68**
	NC	**84.1**	**92.2**					**81.6**	**83.3**				
Shanbhag	C	0.70	3.0	48.5	58.3	0.00	0.03	29.1	42.5	63.2	75.1	0.28	0.45
	NC	100	100					99.7	99.6				
Renyi-Sahoo	C	46.5	57.5	68.7	81.3	0.38	0.60	62.4	56.0	78.6	80.5	0.58	0.58
	NC	92.5	99.4					95.9	98.9				
Yen	C	46.5	57.5	68.7	81.3	0.38	0.60	56.7	52.8	76.1	79.2	0.53	0.55
	NC	92.5	99.4					96.9	99.1				

**Table 3.** Individual and global accuracies obtained for the thresholded indices corresponding to level 3, areas A1 & A2.

**Thresholding Method**	**Class**	**CVA**						**CVA-TEXT-HOMOGENEITY**					

		**Individual Acc. %**		**Overall Acc. %**		**Kappa Coeff.**		**Individual Acc. %**		**Overall Acc. %**		**Kappa Coeff**	

		**A1**	**A2**	**A1**	**A2**	**A1**	**A2**	**A1**	**A2**	**A1**	**A2**	**A1**	**A2**
Ridler—Iter	C	23.3	50.5	49.5	67.9	0.01	0.32	53.4	74.0	68.3	84.6	0.37	0.68
	NC	77.5	81.2					84.4	92.6				
Otsu	C	24.3	50.8	49.7	67.6	0.01	0.32	53.4	74.0	68.3	84.6	0.37	0.68
	NC	76.9	80.34					84.4	92.6				
Kapur	C	2.8	32.8	45.4	68.7	0.06	0.31	18.0	52.3	54.3	78.7	0.11	0.54
	NC	91.1	85.8					93.2	98.7				
Li	C	41.5	57.4	54.0	61.2	0.09	0.21	**76.3**	**82.9**	**75.6**	**81.7**	**0.51**	**0.63**
	NC	67.5	64.2					**74.9**	**80.7**				
Shanbhag	C	15.5	47.9	47.3	70.3	0.03	0.37	0.70	19.6	48.4	65.3	0.00	0.22
	NC	81.4	68.9					99.5	100				
Renyi-Sahoo	C	4.4	39.6	45.2	70.8	0.06	0.36	17.97	52.3	54.3	78.7	0.11	0.54
	NC	89.0	94.5					93.2	98.7				
Yen	C	**4.5**	**40.1**	**45.2**	**71.0**	**0.06**	**0.37**	16.5	50.7	53.7	78.1	0.10	0.52
	NC	**88.8**	**94.4**					93.7	98.8				

**Table 4.** Level 1. Individual and global accuracies obtained for the multisource process as a function of the weights assigned to each index (CVA & Diff. NDVI). Sumative method (Σ), Multiplicative method (Π).

**Combination**	**Class**	**Individual Acc. %**		**Overall Acc. %**		**Kappa Coeff.**		**True Positives Rate**		**False Positives Rate**	

		**Σ**	**Π**	**Σ**	**Π**	**Σ**	**Π**	**Σ**	**Π**	**Σ**	**Π**
1. λ_CVA_ = 0.0, λ_NDVI_ = 1.0	C	65.4	x	78.1	x	0.55	x	0.65	x	0.11	x
	NC	89.2	x								
2. λ_CVA_ = 0.1, λ_NDVI_ = 0.9	C	65.1	-	78.2	-	0.55	-	0.65	-	-	-
	NC	89.7	-								
3. λ_CVA_ = 0.2, λ_NDVI_ = 0.8	C	65.1	-	78.2	-	0.55	-	0.65	-	0.10	-
	NC	89.7	-								
4. λ_CVA_ = 0.3, λ_NDVI_ = 0.7	C	65.6	-	78.3	-	0.56	-	0.65	-	0.10	-
	NC	89.5	-								
5. λ_CVA_ = 0.4, λ_NDVI_ = 0.6	C	67.1	-	79.2	-	0.58	-	0.67	-	0.10	-
	NC	90.0	-								
6. λ_CVA_ = 0.5, λ_NDVI_ = 0.5	C	83.8	-	**86.7**	-	**0.73**	-	0.83	-	0.11	-
	NC	89.2	-								
7. λ_CVA_ = 0.6, λ_NDVI_ = 0.4	C	84.5	81.4	**86.7**	**86.1**	**0.73**	**0.72**	0.84	0.85	0.12	0.12
	NC	88.5	90.2								
8. λ_CVA_ = 0.7, λ_NDVI_ = 0.3	C	84.6	-	86.4	-	0.72	-	0.85	-	0.12	-
	NC	88.0	-								
9. λ_CVA_ = 0.8, λ_NDVI_ = 0.2	C	84.5	-	86.4	-	0.72	-	0.85	-	0.12	-
	NC	88.1	-								
10. λ_CVA_ = 0.9, λ_NDVI_ = 0.1	C	84.7	-	86.4	-	0.72	-	0.85	-	0.12	-
	NC	88.1	-								
11. λ_CVA_ = 1.0, λ_NDVI_ = 0.0	C	85.5	x	86.5	x	0.72	x	0.85	x	0.12	x
	NC	87.4	x								

**Table 5.** Level 2. Individual and overall accuracies obtained for the multisource process as a function of the weights assigned to each index (Diff. & Ratio). Additive method (Σ), Multiplicative method (Π).

**Combination**	**Class**	**Individual Acc. %**		**Overall Acc. %**		**Kappa Coeff.**		**True Positives Rate**		**False Positives Rate**		

		**Σ**	**Π**	**Σ**	**Π**	**Σ**	**Π**	**Σ**	**Π**	**Σ**		**Π**
1. λ_DIFF_ = 0.0, λ_RATIO_ = 1.0	C	76.1	x	85.4	x	0.7	x	0.76	x	0.06	x	
	NC	93.4	x									
2. λ_DIFF_ = 0.1, λ_RATIO_ = 0.9	C	76.5	-	85.5	-	0.71	-	0.76		0.07	-	
	NC	93.3	-									
3. λ_DIFF_ = 0.2, λ_RATIO_ = 0.8	C	76.8	-	85.6	-	0.71	-	0.76	-	0.07	-	
	NC	93.2	-									
4. λ_DIFF_ = 0.3, λ_RATIO_ = 0.7	C	76.9	-	85.7	-	0.71	-	0.77	-	0.07	-	
	NC	93.2	-									
5. λ_DIFF_ = 0.4, λ_RATIO_ = 0.6	C	77.7	-	85.9	-	0.71	-	0.77	-	0.07	-	
	NC	93.0	-									
6. λ_DIFF_ = 0.5, λ_RATIO_ = 0.5	C	83.6	-	87.7	-	0.75	-	0.83	-	0.09	-	
	NC	91.1	-									
7. λ_DIFF_ = 0.6, λ_RATIO_ = 0.4	**C**	**86.4**	**79.5**	**88.3**	**86.2**	**0.76**	**0.72**	0.86	0.8	0.10	0.08	
	**NC**	**90.0**	**92.0**									
8. λ_DIFF_ = 0.7, λ_RATIO_ = 0.3	**C**	**86.5**	-	**88.3**	-	**0.76**	-	0.86	-	0.10	-	
	**NC**	**89.8**	-									
9. λ_DIFF_ = 0.8, λ_RATIO_ = 0.2	**C**	**87.0**	-	**88.3**	-	**0.76**	-	0.87	-	0.10	-	
	**NC**	**89.4**	-									
10. λ_DIFF_ = 0.9, λ_RATIO_ = 0.1	**C**	**87.0**	-	**88.3**	-	**0.76**	-	0.87	-	0.10	-	
	**NC**	**89.5**	-									
11. λ_DIFF_ = 1.0, λ_RATIO_ = 0.0	**C**	**87.0**	x	**88.3**	x	**0.76**	x	0.87	x	0.10	x	
	**NC**	**89.5**	x									
